# Combined Detection of Preoperative Neutrophil-to-Lymphocyte Ratio and CEA as an Independent Prognostic Factor in Nonmetastatic Patients Undergoing Colorectal Cancer Resection Is Superior to NLR or CEA Alone

**DOI:** 10.1155/2017/3809464

**Published:** 2017-06-08

**Authors:** Xiaoli Zhan, Xiaobo Sun, Yonggang Hong, Yuedong Wang, Kefeng Ding

**Affiliations:** ^1^Department of General Surgery, The Second Affiliated Hospital, Zhejiang University College of Medicine, Hangzhou 310009, China; ^2^Department of Laboratory Medicine, Changhai Hospital, 168 Changhai Road, Shanghai 200433, China; ^3^Department of General Surgery, Changhai Hospital, 168 Changhai Road, Shanghai 200433, China; ^4^Department of Medical Oncology, The Second Affiliated Hospital, Zhejiang University College of Medicine, Hangzhou 310009, China

## Abstract

**Objective:**

To explore the role of combined detection of carcinoembryonic antigen (CEA) and neutrophil-to-lymphocyte ratio (NLR) in the prognostic assessment of colorectal cancer (CRC).

**Methods:**

We investigated preoperative NLR and CEA in 125 surgical CRC patients, determined the patients' thresholds by receiver operating characteristic (ROC) curve analysis, and assessed their prognostic values by Kaplan–Meier curve and Cox regression models. In addition, we used nomograms of several risk factors to evaluate the risk in survival and predictive accuracy by using Harrell's concordance index (*c*-index).

**Results:**

Results of multivariate analysis showed high NLR, high CEA, and high COCN (combination of CEA and NLR) were significantly correlated with decreased disease-free survival (DFS) [HR: 2.229, 95% CI: 1.012–4.911, and *P* = 0.047; HR: 3.652, 95% CI: 1.630–8.179, and *P* = 0.002; HR: 3.139, 95% CI: 1.800–5.472, and *P* < 0.001]. But high CEA and COCN remained significant only for decreased overall survival (OS) [HR: 3.713, 95% CI: 1.396–9.873, and *P* = 0.009; HR: 3.106, 95% CI: 1.576–6.123, and *P* = 0.001]. High NLR showed higher mortality rates with worse OS (*P* = 0.058), and nomograms containing NLR improved the predictive accuracy. Area under the curve of COCN was higher than that of CEA or NLR.

**Conclusion:**

COCN acts as a better independent prognostic biomarker of CRC than NLR or CEA alone.

## 1. Introduction

Colorectal cancer (CRC) is one of the most frequently diagnosed tumors and is the fourth leading cause of cancer-related deaths worldwide, with an annual incidence of 148,000 new cases [1]. Overall survival (OS) of CRC patients is poor, and more than one-third of CRC patients die within 5 years [2]. Although the 5-year OS of CRC patients has been improved remarkably in recent years owing to the advances in surgical techniques and other therapies, 40–50% of patients who underwent colorectal resection developed recurrences or died due to metastatic disease [3, 4].

Inflammatory response plays a key role in the survival of cancer patients. Several recent studies have demonstrated that systemic inflammatory response (SIR) markers, including C-reactive protein (CRP) [5], Glasgow prognostic score (GPS) [6], platelet-to-lymphocyte ratio (PLR), and lymphocyte-to-monocyte ratio (LMR) [7], are correlated with poor survival rates of CRC and many other cancers [5, 8]. The neutrophil-to-lymphocyte ratio (NLR) which is considered as one of the SIR markers has been reported to be associated with the prognosis in patients with various types of cancer [9–12]. Few recent studies have reported the role of NLR as a prognostic factor for CRC patients [5, 8]. However, most of these studies focused on the prognostic role of advanced or metastatic CRC [13–17], and few studies have investigated the prognostic value of NLR in resectable stages II and III primary CRC. Specifically, there were no studies till date that have explored the prognostic role of combined detection of NLR and CEA in patients undergoing colorectal cancer resection and compared the combination with NLR or CEA alone.

It is increasingly recognized that survival of cancer patients was determined not only by the host SIR but also by tumor characteristics [17]. Carcinoembryonic antigen (CEA) is a widely used tumor-related marker for prognostic prediction of CRC patients [2, 8]. We therefore hypothesize that identifying parameters reflecting both tumor characteristics and host SIR may be a better approach for predicting patient survival, and COCN (combination of CEA and NLR) may be a better biomarker in the prognostic assessment of CRC.

Hence, in the present study we first evaluated the prognostic utility of NLR or CEA alone in patients undergoing surgery for CRC and then explored the prognostic value of COCN, a novel inflammation-based prognostic system with tumor characteristics, in an attempt to provide experimental clues for better prediction of CRC prognosis in patients.

## 2. Materials and Methods

### 2.1. Patient Selection

One hundred and sixty patients were included in this study with pathologically confirmed stages II and III CRC without distant metastasis or local recurrence who received surgical resection in Changhai Hospital (Shanghai, China) between March 2013 and October 2014. All patients received curative resection and the surgical procedures were performed by the same surgical team of the same department in all patients. The exclusion criteria included patients who were presented with clinical signs of systemic inflammation or infection, hematological diseases, evidence of hyperpyrexia, enterobrosis, onset of intestinal obstruction during hospitalization, or a history of other malignancies. All the procedures were performed in accordance with the ethical standards of the institutional and/or national research committee and with the Helsinki declaration and were approved by the Ethics Committee of Changhai Hospital with the permit number of 2012B0076, and informed consent was obtained from all included patients.

### 2.2. Clinical Data and Laboratory Methods

Neutrophil count, lymphocyte count, red blood cell (RBC), platelet count, fibrinogen (Fib), CEA, and carbohydrate antigen 199 (CA199) were measured within 3 days prior to the surgery as part of the routine preoperative workup in these patients. Full blood count (FBC) was analyzed with a Sysmex XN-9000 automated hematology analyzer (Sysmex Corporation, Kobe, Tokyo, Japan). Fib was measured with a Sysmex CS-5100 automatic coagulation analyzer (Sysmex UK Ltd., Milton Keynes, UK). CEA and CA199 were conducted by Roche Elecsys 2010 Chemistry Analyzer (Basel, Switzerland). NLR or PLR was calculated by dividing the absolute number of neutrophils or platelets by the absolute number of lymphocytes, respectively.

The clinical data were collected via the hospital information system, which included age, gender, smoking, cancer site, tumor stage, histological class, differentiation, tumor size, and family histology. Tumor staging was performed according to the tumor-node-metastasis (TNM) classification of the American Joint Committee on Cancer (AJCC, 7th edition).

### 2.3. Survival and Follow-Up

All 160 patients were put on a regular follow-up program on the outpatient basis for every 3–6 months for the first 2 years and every 6 months for the next 3 years, which included physical examination, CEA and CA199 test, chest X-ray, abdominal ultrasonography, or abdominal CT and colonoscopy every 1–3 years. Of them, 35 patients were excluded from the study due to loss of follow-up for various reasons.

Recurrence was detected by a combination of imaging studies and tumor markers, such as CEA and CA199, and finally was confirmed by pathological examination [2]. OS was calculated from the date of diagnosis to the date of death or last follow-up visit in survivors (23) [18]. Disease-free survival (DFS) was measured from the date of surgery to the date of disease recurrence or the date of last follow-up visit in patients without recurrences [18].

### 2.4. Statistical Analysis

The cut-off values of NLR, PLR, Fib, CA199, CEA, and RBC were determined by receiver operating characteristic (ROC) curve analysis. According to each cut-off value, patients were divided into two groups. Furthermore, according to COCN score as shown in Table 1, patients were assigned into three groups. Differences in clinicopathological characteristics as grouped by NLR and CEA were compared using the Pearson *χ*^2^ test or Fisher's exact test. Survival analysis was performed using the Kaplan–Meier method and Cox proportional hazard model. The variates with significant differences were identified from the univariate analysis and were selected for forward multivariate Cox regression survival analysis. Differences were estimated by log-rank test. Statistical significance was set at a level of *P* < 0.05. All analyses were performed using SPSS software 21.0 (SPSS Inc., Chicago, USA), and nomogram was explored by R version 3.0.0 software (Institute for Statistics and Mathematics, Vienna, Austria). The optimal thresholds were selected using R package MAXSTAT [19].

## 3. Results

### 3.1. ROC Curves of CEA and NLR for Both DFS and OS

**Figure 1:**
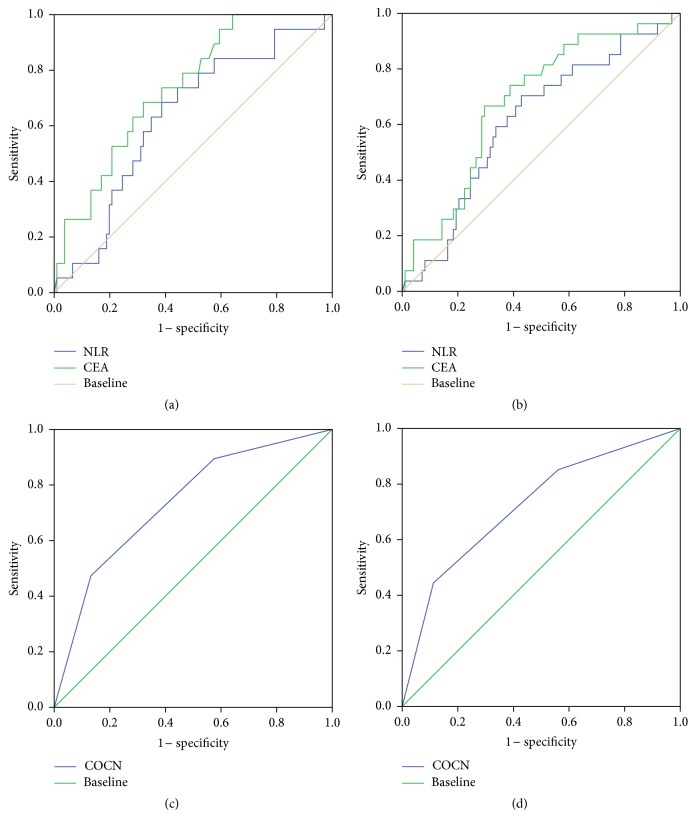
Receiver operating characteristics curve analysis of NLR, CEA, and COCN in CRC patients. ROC curve analysis of NLR and CEA for OS (a) and DFS (b). ROC curve analysis of COCN for OS (c) and DFS (d).

Using ROC analysis, the cut-off value of NLR was calculated based on which patients were assigned to a high NLR (≥2.43) group and low NLR (<2.43) group. Area under the curve (AUC) was 0.605 (95% CI: 0.487 to 0.723) for DFS and 0.619 (95% CI: 0.487 to 0.751) for OS (Figures 1(a) and 1(b)). A cut-off value of 2.43 was chosen as an optimal NLR value for evaluating DFS and OS. This value showed a 0.688 sensitivity and 0.606 specificity for OS and 0.630 sensitivity and 0.622 specificity for DFS. Similarly, based on ROC analysis, a cut-off value of 5 was used for CEA (ng/mL) in our study. Similarly, ROC analysis was applied to PLR, Fib, CA199, and RBC, which showed cut-off values of 113.5, 3.30 (g/mL), 7.42 (U/mL), and 4.43 (×10^12^/mL), respectively.

### 3.2. Correlations of NLR and CEA with Clinicopathological Factors

Correlations of NLR and CEA with various clinicopathological factors including Fib, CA199, PLR, and other clinicopathological characteristics were analyzed. As shown in Table 2, high NLR was significantly correlated with cancer site, tumor differentiation, and PLR, while there was no significant correlation observed between high NLR and other factors. In addition, high CEA was correlated with Fib and CA199 but not with other factors.

### 3.3. Correlations of Clinicopathological Factors with OS and DFS

**Figure 2:**
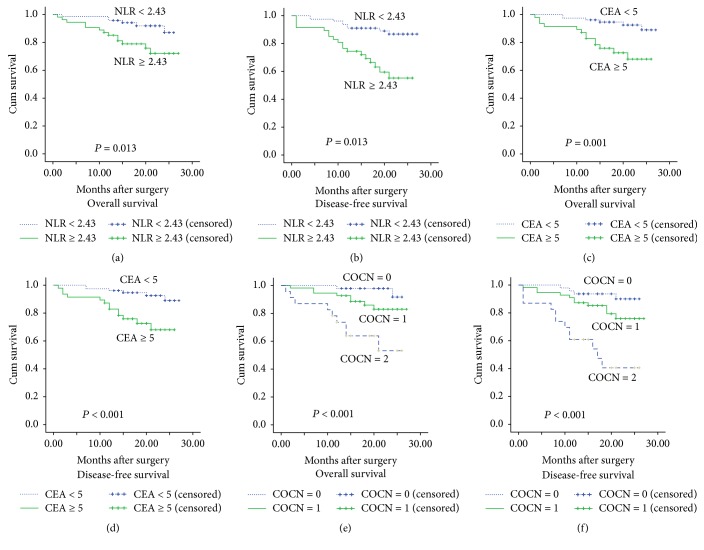
Kaplan–Meier survival curves for overall survival (OS) and disease-free survival (DFS) in 125 patients undergoing primary colorectal cancer resection according to their NLR and CEA levels. (a) OS according to NLR. (b) DFS according to NLR. (c) OS according to CEA levels. (d) DFS according to CEA levels. (e) OS according to COCN. (f) DFS according to COCN. Patients with neither NLR ≥ 2.43 nor CEA ≥ 5 were assigned as COCN = 0, with NLR ≥ 2.43 or CEA ≥ 5 as COCN = 1 and with NLR ≥ 2.43 and CEA ≥ 5 as COCN = 2.

Correlations of clinicopathological factors with OS and DFS were analyzed by using univariate and multivariate analyses. The result of univariate analysis showed that tumor stage, NLR, CEA, and COCN were all related to OS and DFS (Table 3). The result of multivariate analysis showed that high CEA (≥5) was strongly correlated with decreased DFS (HR: 3.652, 95% CI: 1.630–8.179, and *P* = 0.002) and OS (HR: 3.713, 95% CI: 1.396–9.873, and *P* = 0.009). High NLR (≥2.43) was correlated with worse DFS (HR: 2.229, 95% CI: 1.012–4.911, and *P* = 0.047) and the results were presented in Figures 2(a), 2(b), 2(c), and 2(d). However, high NLR (≥2.43) showed only a strong trend with lower OS (HR: 2.571, 95% CI: 0.968–6.832, and *P* = 0.058) (Table 4).

### 3.4. Nomogram Analysis with or without NLR for OS and DFS

**Figure 3:**
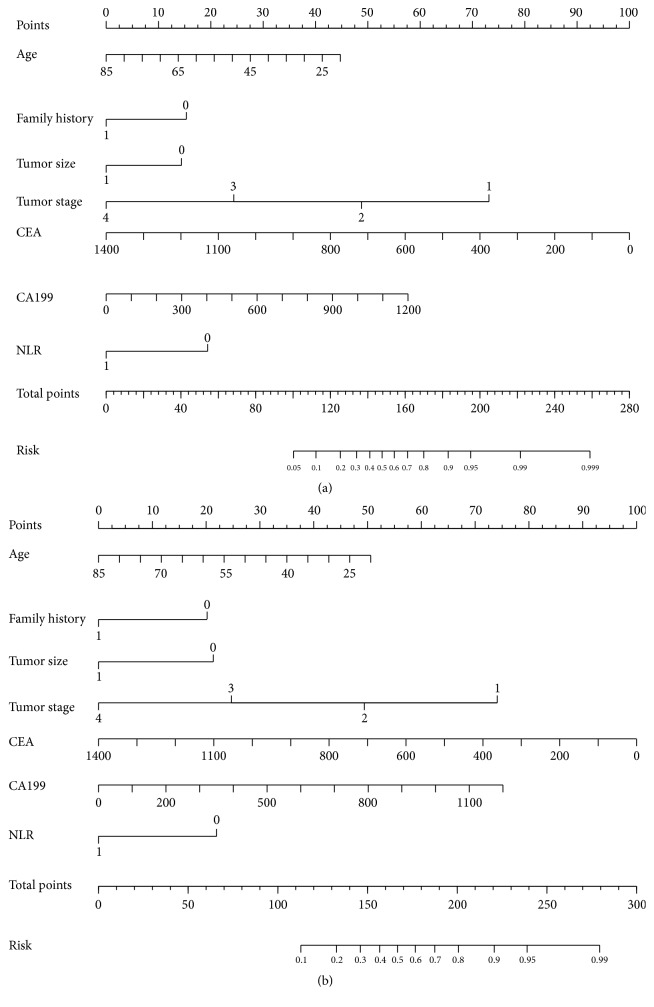
Nomograms that predict the probability of NLR in OS (a) and DFS (b).

To evaluate the prognostic value of NLR in CRC, nomogram analysis for OS and DFS was performed (Figure 3). The concordance index (*C*-index) of the nomogram with NLR for OS and DFS was 0.810 and 0.802, respectively. However, the *C*-index of the nomogram without NLR for OS and DFS was only 0.656 and 0.688, respectively. These results suggested that the *C*-index of the nomogram with NLR may better predict clinical outcomes in CRC patients than without NLR.

### 3.5. COCN Is a Superior Prognostic Biomarker

As we showed above, NLR and CEA were shown to be independent prognostic biomarkers in CRC patients, but whether COCN had the same efficacy still remained unclear. Therefore, we studied the value of COCN in our patients using Kaplan–Meier method and Cox regression model. We first performed a univariate Cox regression survival analysis and then selected the variates with significant differences identified from the univariate analysis for multivariate Cox regression survival analysis. For COCN, the result of univariate Cox regression showed that survival was different between the three groups (COCN = 1,0, 2) of patients (*P* < 0.05) (Table 3), and the multivariate Cox regression survival analysis showed that COCN was an independent prognostic factor (Table 4). The results of Kaplan–Meier method was presented in Figures 2(e) and 2(f). Finally, ROC of COCN was used to assess the prognostic value of COCN. The result showed that AUC of COCN was 0.737 (95% CI: 0.616 to 0.857) for OS (Figure 1(c)) and 0.722 (95% CI: 0.611 to 0.833) for DFS (Figure 1(d)), which was higher than that of NLR for OS (0.619) and DFS (0.605) (Figures 1(a) and 1(b)). At the same time, AUC of COCN was 0.722 for DFS, which were also higher than AUC of CEA for DFS (0.688) (Figure 1(b)). These results implied that COCN acts as a significant prognostic biomarker that can be superior to either NLR or CEA alone.

## 4. Discussion

In the present study, we investigated the correlations among SIR, clinicopathological characteristics, and survival in patients with primary CRC undergoing surgical resection. Our results demonstrated that high NLR was correlated with cancer site, tumor differentiation, and PLR, while high CEA was correlated with Fib and CA199 level. In addition, CEA and NLR were both independent prognostic factors associated with DFS in patients with primary CRC. CEA was an independent positive prognostic marker for OS in CRC, while high NLR showed a significant trend with lower OS rates (*P* = 0.058). Moreover, the current study for the first time demonstrated that COCN was more effective candidate prognostic biomarker in patients undergoing surgical resection of CRC than NLR or CEA alone.

Based on the results of ROC analysis, we chose a cut-off value of 2.43 for NLR because it yielded the highest sensitivity and specificity. Cut-off value of our study was approximately equal to 2.5 as per Shibutani et al. [20] study and 2.4 as per Neofytou et al. [21] study in CRC patients, and a little lower than 2.57 was reported in Yao et al. [22] study of breast cancer. Therefore, a cut-off value of 2.43 for NLR is considered as an acceptable value in several types of cancer.

Our study demonstrated that elevated NLR was an independent prognostic factor for poor survival of CRC patients. This finding was consistent with previous reports regarding CRC. For instance, a study by Shin et al. [23] demonstrated that preoperative NLR could predict survival rate in resectable patients with stage T1-2N0 CRC. Kubo et al. [24] also demonstrated that the pre- and postoperative NLR were both considered to be good predictor for the long-time survival in CRC patients. These studies together with ours suggest that high NLR values have a prognostic significance in CRC patients.

Although the reason for association between high NLR and poor prognosis is very complex and remains to be elucidated, there are several potential mechanisms. Patients with elevated NLR showed a relative lymphopenia and a high-circulating neutrophil level [25]. Neutrophils are known to play a key role in all stages of tumor progression by (1) producing a number of ligands and secretion of matrix metalloproteinases (MMPs), thus inducing tumor cell proliferation and invasion [26]; (2) releasing proangiogenic chemokines and other cytokines to promote tumor vascular formation [27, 28]; and (3) interacting with T cell, thus affecting tumor cell proliferation, angiogenesis, and metastasis [29]. Taken together, neutrophils may act as a tumor-promoting factor in various stages of cancer [30]. In contrast, lymphocytes could inhibit the proliferation and metastasis of tumor cells by participating in cytotoxic cell death and inducing the secretion of cytokines against tumor formation [20]. Given the tumor-promoting role of neutrophils and the antitumor effect of lymphocytes, elevation of NLR may reflect the increased protumor activity of neutrophils or the reduced antitumor immune responses by lymphocytes, consequently resulting in poorer survival of patients with elevated NLR. This might explain as to why patients with elevated NLR had a significantly poorer prognosis in the the present study.

Our research demonstrated NLR and preoperative CEA as independent prognostic factors in predicting the survival rates, and our results were similar to the previous reports [31–33]. Toiyama et al. [31] suggested that elevated CEA was a predictor of poor OS in rectal cancer patients who were treated with preoperative radiotherapy and chemotherapy. Thirunavukarasu et al. [33] confirmed that serum CEA was an independent prognostic marker in CRC patients with a mean follow-up period of 27 months. Graham et al. [32] considered that testing the CEA level of CRC patients was the most cost-effective for predicting the postoperative recurrences. Because of the prognostic usefulness of CEA, the American Society of Clinical Oncology (ASCO) and the European Society for Medical Oncology (ESMO) recommended CEA level to be considered as a golden follow-up standard after CRC therapy [34, 35].

We did not find significant correlations of the PLR and clinical characteristics with DFS or OS. This is not consistent with the result reported by Kwon et al. [36], who found that high PLR was independently associated with poorer OS and DFS. There are several reasons associated with differences in the results. One reason might be due to the different cut-off values used in this study. Presently, there is no consensus on cut-off values of PLR and the optimal cut-off value for PLR was determined either by ROC analysis as was the case with our study or according to the median of PLR. Another possible reason was due to tumor specificity and underlying genetic and biological differences between distinct patient cohorts. Finally, analyzers from different manufacturers could also cause differences in the PLR levels [37]. All these reasons put together may lead to the differences in the results as observed in these studies. Therefore, it is difficult to make a fair comparison between these studies and further studies are required to validate these possible reasons.

A more recent study reported that tumor progression was significantly correlated with tumor characteristics and host SIR [38]. Therefore, identifying parameters that reflect both tumor characteristics and host SIR provides a better prognostic value. Meanwhile, high CEA levels were reported to be related to the invasion and metastasis of tumor cells [34] and elevated NLR was considered to be a main factor of host SIR to tumors [39]. Our results further showed that CEA and NLR were both independent prognostic markers in CRC patients. We therefore hypothesized that combined detection of CEA and NLR may have a more important prognostic value in CRC. As shown in Table 4, COCN was an independent prognostic biomarker. Our study result was similar to the study results of He et al. [40], who supported that NLR in combination with CEA may provide a useful prognostic value in metastatic CRC patients. Further to this, our study for the first time indicated useful prognostic value of COCN in the primary resected CRC patients (Table 4), but it was also considered to be a better predictor of postoperative prognosis than NLR or CEA alone.

Our study demonstrated that COCN was correlated with survival of patients with resected primary CRC and supported the categorization of CRC patients into groups as favorable or poor prognosis based on the combined detection of inflammation-based and tumor-related factors. Highlights of the present study included the use of a uniform approach for patient assessment. On the other hand, COCN is easy to measure because of its low cost and convenience. Additionally, compared with NLR and CEA alone, COCN not only reflects the inflammatory and immune status of the patient but also represents the tumor characteristics and therefore is considered as a good prognostic marker in primary CRC patients.

Our study has few limitations. Firstly, selection bias could not be completely excluded due to the single-institutional and retrospective nature of the study. Secondly, the number of patients was small and the follow-up duration was relatively short. Finally, standardization of all clinical assays was another important problem. Although NLR and CEA are easy to measure, their utility might be affected by several factors and, therefore, more standard criteria need to be considered. Despite these limitations, still our study suggests that COCN was proved to be a better independent prognostic biomarker of CRC than NLR or CEA alone. Future studies are needed to prospectively validate the prognostic usefulness of COCN in CRC patients and assess the predictive nature of COCN to guide CRC therapy as well.

In summary, our research showed COCN to be an independent prognostic biomarker for CRC. Owing to the convenience, low cost, and high prognostic value, COCN may serve as a good biomarker in optimizing patient selection for further treatment, predicting OS and DFS, and decreasing the morbidity in patients undergoing curative resection for primary CRC.

## Figures and Tables

**Table 1:** Prognostic scores of NLR, CEA, and COCN.

Scoring system	Score
NLR	
≥2.43	1
<2.43	0
CEA	
≥5 ng/mL	1
<5 ng/mL	0
Combination of CEA and NLR (COCN)	
NLR ≥ 2.43 and CEA ≥ 5 ng/mL	2
NLR ≥ 2.43 or CEA ≥ 5 ng/mL	1
Neither NLR ≥ 2.43 nor CEA ≥ 5 ng/mL	0

NLR = neutrophil-to-lymphocyte ratio; CEA = carcinoembryonic antigen.

**Table 2:** Characteristics of the 125 patients grouped by NLR and CEA.

Variables	Sum (%)	NLR			CEA (ng/mL)		
		≥2.43	<2.43	*P* value	≥5	<5	*P* value
Age (year)							
<65	70 (56)	32	38	0.587	23	47	0.265
≥65	55 (44)	22	33		24	31	
Gender							
Female	52 (0.42)	24	28	0.588	22	30	0.454
Male	73 (0.58)	30	43		25	48	
Smoking							
Yes	116 (0.92)	50	66	1.000	45	71	0.481
No	9 (0.08)	4	5		2	7	
Family history							
No	118 (0.944)	51	67	1.000	45	73	0.710
Yes	7 (0.056)	3	4		2	5	
Cancer site							
Rectum	88 (0.7)	31	57	0.006	32	56	0.689
Colon	37 (0.3)	23	14		15	22	
Histologic class							
AC	120 (0.96)	50	70	0.165	45	75	1.000
NAC	5 (0.04)	4	1		2	3	
Differentiation							
Well/moderate	4 (0.03)	4	0	0.033	1	3	0.663
Poor	121 (0.97)	50	71		46	75	
Tumor stage							
II	56 (0.452)	20	36	0.149	18	38	0.272
III	68 (0.548)	34	35		29	40	
Tumor size (cm)							
<5	116 (0.92)	45	64	0.289	38	71	0.165
≥5	9 (0.08)	9	7		9	7	
Fib (g/mL)							
<3.30	47 (0.38)	19	29	0.580	11	37	0.008
≥3.30	76 (0.62)	35	42		36	41	
RBC (×10^12^/mL)							
<4.43	77 (0.62)	38	39	0.095	28	49	0.850
≥4.43	48 (0.38)	16	32		19	29	
PLR							
<113.5	48 (0.38)	4	44	<0.001	18	30	1.000
≥113.5	77 (0.62)	50	27		29	48	
NLR							
<2.43	71 (0.57)	—	—	—	24	47	0.354
≥2.43	54 (0.43)	—	—		23	31	
CEA (ng/mL)							
<5	78 (0.72)	31	47	0.206	—	—	—
≥5	47 (0.38)	23	24		—	—	
CA199 (U/mL)							
<7.42	50 (0.40)	18	32	0.202	13	37	0.038
≥7.42	75 (0.60)	36	39		34	41	

Fib = fibrinogen; RBC = red blood cell; PLR = platelet-to-lymphocyte ratio; NLR = neutrophil-to-lymphocyte ratio; CEA = carcinoembryonic antigen; CA199 = carbohydrate antigen 199; COCN = combination of CEA and NLR; AC = adenocarcinoma; NAC = nonadenocarcinoma.

**Table 3:** Univariate Cox regression survival analysis for all CRC patients undergoing surgery (*n* = 125).

Variables	OS			DFS		
	HR	95% CI	*P* value	HR	95% CI	*P* value
Age (≥65 versus <65)	1.469	0.596–3.622	0.404	1.405	0.660–2.991	0.378
Gender (male versus female)	1.952	0.703–5.424	0.191	1.692	0.740–3.865	0.212
Smoking	2.745	0.798–9.451	0.109	1.953	0.587–6.493	0.275
Family history	1.618	0.372–7.040	0.521	1.755	0.527–5.844	0.360
Cancer site	1.264	0.477–3.345	0.637	1.370	0.612–3.064	0.444
Histologic class	1.492	0.199–11.204	0.698	1.059	0.143–7.813	0.956
Differentiation	21.018	0–3707867.689	0.621	0.697	0.094–5.146	0.723
Tumor stage	4.491	1.308–15.418	0.017	3.049	1.231–7.557	0.016
Tumor size (≥5 cm versus <5 cm)	1.923	0.634–5.827	0.248	2.069	0.833–5.141	0.117
Fib (≥3.30 g/mL versus <3.30 g/mL)	1.092	0.429–2.777	0.853	1.263	0.567–2.812	0.568
RBC						
≥4.43 versus <4.43 (×10^12^/mL)	1.460	0.593–3.596	0.410	1.538	0.723–3.275	0.264
PLR (≥113.5 versus <113.5)	2.656	0.881–8.010	0.083	2.048	0.865–4.846	0.103
NLR (≥2.43 versus <2.43)	3.193	1.213–8.405	0.019	2.582	1.182–5.644	0.017
CEA (≥5 ng/mL versus <5 ng/mL)	4.351	1.645–11.509	0.003	4.023	1.803–8.974	0.001
CA199						
≥7.42 U/mL versus <5 U/mL	2.496	0.828–7.520	0.104	1.602	0.701–3.660	0.264

AC = adenocarcinoma; NAC = nonadenocarcinoma; Fib = fibrinogen; RBC = red blood cell; PLR = platelet-to-lymphocyte ratio; NLR = neutrophil-to-lymphocyte ratio; CEA = carcinoembryonic antigen; CA199 = carbohydrate antigen 199; COCN = combination of CEA and NLR; HR = hazard ratio; OS = overall survival; DFS = disease-free survival.

**Table 4:** Multivariate Cox regression survival analysis for all patients undergoing surgery (*n* = 125).

Variables	OS			DFS		
	HR	95% CI	*P* value	HR	95% CI	*P* value
NLR (≥2.43 versus <2.43)	2.571	0.968–6.832	0.058	2.229	1.012–4.911	0.047
CEA (≥5 ng/mL versus <5 ng/mL)	3.713	1.396–9.873	0.009	3.652	1.630–8.179	0.002
COCN (0,1, 2)	3.106	1.576–6.123	0.001	3.139	1.800–5.472	<0.001
Tumor stage	3.384	0.972–11.778	0.055	2.449	0.978–6.131	0.056

NLR = neutrophil-to-lymphocyte ratio; CEA = carcinoembryonic antigen; COCN = combination of CEA and NLR; HR = hazard ratio; OS = overall survival; DFS = disease-free survival.
